# Synchrony of Caresses: Does Affective Touch Help Infants to Detect Body-Related Visual–Tactile Synchrony?

**DOI:** 10.3389/fpsyg.2019.02944

**Published:** 2020-01-09

**Authors:** Letizia Della Longa, Maria Laura Filippetti, Danica Dragovic, Teresa Farroni

**Affiliations:** ^1^Department of Developmental Psychology and Socialization, University of Padua, Padua, Italy; ^2^Centre for Brain Science, Department of Psychology, Faculty of Science and Health, University of Essex, Essex, United Kingdom; ^3^Department of Pediatric Unit, Hospital of Monfalcone, Monfalcone, Italy

**Keywords:** body awareness, multisensory, affective touch, visual preference, infancy

## Abstract

Bodily self-awareness, that is the ability to sense and recognize our body as our own, involves the encoding and integration of a wide range of multisensory and motor signals. Infants’ abilities to detect synchrony and bind together sensory information in time and space critically contribute to the process of gradual bodily self-awareness. In particular, early tactile experiences may have a crucial role in promoting self-other differentiation and developing bodily self-awareness. More specifically affective touch, slow and gentle touch linked to the neurophysiologically specialized system of C-tactile afferents, provides both information about the body from within (interoception) and outside (exteroception), suggesting it may be a key component contributing to the experience of bodily self-awareness. The present study aimed to investigate the role of affective touch in the formation and modulation of body perception from the earliest stages of life. Using a preferential looking task, 5-month-old infants were presented with synchronous and asynchronous visuo–tactile body-related stimuli. The socio-affective valence of the tactile stimuli was manipulated by means of the velocity [CT-optimal (slow) touch vs. CT-suboptimal (fast) touch] and the source of touch (human hand vs. brush). For the first time, we show that only infants that were stroked using a brush at slow velocity displayed a preference for the visual–tactile synchronous video, suggesting that CT-optimal touch might help infants to detect body-related visual–tactile synchrony, independently from the source of touch. Our results are in line with findings from adults and indicate that affective touch might have a critical role in the early development of bodily self-awareness.

## Introduction

Our body is the mean by which we engage with the surrounding physical and social world, providing the background condition that enables perception and action (Riva, 2018). Therefore, being able to represent one’s own and others’ bodies is fundamental to human perception, cognition, and behavior (Slaughter and Brownell, 2012). Bodily self-awareness, that is the ability to sense and recognize our body as separate from the environment (Craig, 2002), gradually arises via active process of multisensory perception and exploration, allowing to bind together external sensory information (exteroception; e.g., vision and audition) and internal bodily information (proprioception, which is the sense of body position from input of muscles and joints, and interoception, which refers to the physiological condition of the body originating from visceral sensation; Craig, 2003).

Spatio-temporal correlation or synchrony plays a fundamental role to the effective processing of multisensory information. Indeed, our brain selectively combines related signals across the continuous stream of multisensory inputs based on spatial and temporal co-occurrence (Parise and Ernst, 2016). Adult studies explored the role of multisensory integration in the experience of bodily self-awareness based on the induction of illusory states of body ownership (i.e., the feeling that “my body” belongs to me; Gallagher, 2000). By manipulating the synchrony of visual–tactile input, it is possible to induce an illusory feeling of ownership for an artificial hand (the rubber hand illusion paradigm; Botvinick and Cohen, 1998), for another person’s face (enfacement illusion; Tsakiris, 2008), as well as the whole body (e.g., full-body illusion; Petkova and Ehrsson, 2008). These perceptual illusions consist in manipulating the multisensory information related to the body to create an illusory self-attribution of the external body part, i.e., feeling a rubber hand as part of the own body or perceiving another person’s face as being more similar to one’s own face.

These studies have extensively shown that multisensory signals contribute to bodily self-awareness in adults; however, the ability to perceive spatio-temporal synchrony through the body lies also at the core of the development of bodily self-awareness from infancy onward. Developmental studies have indeed demonstrated that infants differentiate sensations originating from within and outside the body, by showing the ability to discriminate visual-proprioceptive (Bahrick and Watson, 1985; Rochat and Morgan, 1995; Morgan and Rochat, 1997), visual–tactile (Zmyj et al., 2011; Filippetti et al., 2013; Filippetti et al., 2015), and visual–interoceptive contingencies (Maister et al., 2017). This suggests that implicit bodily self-awareness is based on multisensory integration of bodily signals and early detection of synchrony between vision and sensory feedback from the body. Zmyj et al. (2011) have shown that 7- and 10-month-old infants look longer to a video displaying doll’s legs simultaneously touched with their own legs, compared to asynchronous visual–tactile stimulation of the same legs. Filippetti et al. (2013) further demonstrated that even 1-day newborns display a visual preference to visual–tactile synchrony. Interestingly, both studies suggest that top-down constraints modulate sensitivity to multisensory synchronous stimulation as infants did not show any visual preference between synchronous and asynchronous conditions when the reference to the body was disrupted by substituting body parts with objects (e.g., wood sticks instead of doll’s legs; Zmyj et al., 2011) or presenting an inverted face instead of an upright face (Filippetti et al., 2013). These results indicate that the ability to detect multisensory synchrony provides infants with crucial information for perceiving their own body as the subject of a given experience, and thus developing an early sense of bodily self-awareness.

More recently it became apparent that beyond exteroceptive cues, signals arising from within the body itself are critical for bodily self-awareness (Tsakiris, 2017). Adult research begun to address the impact of interoceptive processing on the modulation of bodily self-awareness by emphasizing the primary role of the representation of the body from within (e.g., Tsakiris et al., 2011; Suzuki et al., 2013) which may provide a coherent and stable representation of the physiological condition of the body in response to external changes, reflecting the need of balance between adaptability and stability (Tsakiris, 2017; Palmer and Tsakiris, 2018). Specifically, affective touch has recently gained more attention for its special properties of being invested by both exteroceptive and interoceptive qualities (Fotopoulou and Tsakiris, 2017; Crucianelli and Filippetti, 2018). As a result, it has been suggested that affective touch plays a fundamental role in homeostatic regulation, building an image of the physical self as a feeling entity (Craig, 2002; Olausson et al., 2002; Bjornsdotter et al., 2009), and constitutes a link between perception of external objects and perception of the own body, by providing at the same time information about the external world and the body itself (Fotopoulou and Tsakiris, 2017). Adult studies have shown that affective touch, known to elicit interoceptive feelings of pleasantness, influences bodily illusion and self-face recognition more than non-affective touch (Crucianelli et al., 2013; Lloyd et al., 2013; van Stralen et al., 2014; Panagiotopoulou et al., 2017), suggesting that this type of tactile experience affects the modulation of body boundaries promoting self-other differentiation and bodily self-awareness (Fotopoulou and Tsakiris, 2017).

More specifically, affective touch refers to a separate dimension of tactile stimulation, distinct from sensory–discriminative touch on the basis of afferent responses, electrophysiological properties, axonal projections, and brain activation (McGlone et al., 2014). Neurophysiological studies show that positive and affective components of touch are conveyed via C-tactile fibers, a class of low-threshold, unmyelinated afferents that are present only in the hairy skin of mammals (Morrison et al., 2010) and that preferentially respond to gentle stroking delivered at slow velocity (range between 1 and 10 cm/s) within skin-like temperatures (Ackerley et al., 2014) showing an inverted U-shape between stroking velocity and firing rate (Löken et al., 2009). Importantly, increased firing frequency of C-tactile fibers correlates with high ratings of touch pleasantness, suggesting that these afferents are critically involved in processing pleasant aspects of touch (Löken et al., 2009). Moreover, C-tactile fibers are distinct from the myelinated tactile fibers that code for discriminative touch, as they take a distinct ascending pathway from the periphery to the posterior insula, the secondary somatosensory cortex, and an extended network of brain regions known to be involved in social perception, including the posterior superior temporal sulcus, the medial prefrontal cortex, and the anterior cingulate cortex (Olausson et al., 2002; Gordon et al., 2013; Bjornsdotter et al., 2014). In particular, the insula integrates inputs from multiple sensory modalities and limbic cortical regions, which are involved in processing emotional and rewarding stimuli, suggesting it is critically involved in maintaining the organism’s homeostasis and creating an interoceptive representation of the body (Craig, 2003). Such representation lies at the core of the formation of subjective feelings and bodily self-awareness (Craig, 2009). Neuroimaging studies reported insular cortex as a primary target for C-tactile fibers (Olausson et al., 2002; Bjornsdotter et al., 2010; Davidovic et al., 2018) and the same brain region seems to be the critical lesion site for neurological disturbances in the sense of body ownership (Baier and Karnath, 2008), showing evidence of the involvement of insular cortex in bodily self-awareness. Importantly, developmental studies show that this brain region is responsive to affective touch within the first weeks of life (Jönsson et al., 2018; Tuulari et al., 2019), thus suggesting that affective touch may shape brain development by promoting cognitive and social functioning from the earliest stages of development. Indeed, different studies showed evidence of the importance of affective touch in regulating infants’ behavioral and physiological reactivity to stress during periods of maternal deprivation (Stack and Muir, 1992; Feldman et al., 2010), shaping affiliative behaviors and social bonding (Feldman, 2011; Walker and McGlone, 2013), acting as a reinforcer for maintaining infants’ eye-contact and smiling (Pelaez-Nogueras et al., 1996), and facilitating learning of contingent social information (Della Longa et al., 2019). However, there is a lack of knowledge about the potential role of affective touch on the development of bodily self-awareness.

Considering the neurophysiological characteristics of affective touch and evidence from adult research (Crucianelli et al., 2013; van Stralen et al., 2014; Panagiotopoulou et al., 2017), in the present study we aimed to investigate whether affective touch may promote implicit bodily self-awareness in early infancy, by facilitating the detection of body-related visual–tactile contingencies. More specifically, we built on a previous visual preference paradigm developed by Filippetti et al. (2016). In this study, 5-month-old infants were presented with two side-by-side videos of a baby’s face being stroked on the cheek. One video was time delayed by 3 s compared to the other. During video presentation, infants were touched on their own cheek such as this tactile stimulation was perfectly synchronous with one video and asynchronous relatively to the other video. Results revealed that infants looked significantly longer toward the video that matched exactly the tactile stimulation that they perceived, suggesting infants’ ability to detect synchronous visual–tactile stimulation referred to their own body. Critically, in the present study we propose to take a step further by manipulating the tactile stimulation to within the optimal vs. suboptimal range for activating C-tactile fibers. We hypothesize that affective touch performed at slow velocity, optimal for activating C-tactile fibers, would be more effective in directing infants’ attention to body-related synchronous cue, compared to fast touch that it is not optimal for activating C-tactile fibers.

In the present study, we also manipulated the source of touch, by comparing human hand touch with tactile stimulation delivered by an inanimate object (a brush) as we were interested in investigating whether there is a difference in how infants process skin to skin vs. object contact. Previous studies in adult population demonstrated that C-tactile fibers activation is mediated by both mechanical and thermal properties of the tactile stimulus and their firing frequency correlates with hedonic ratings only for skin temperature (Ackerley et al., 2014). Moreover, gentle stroking with a hand elicits larger responses in somatosensory areas and posterior insula compared to tapping with a velvet stick, suggesting that direct interpersonal contact is processed differently from touch applied through inanimate objects (Kress et al., 2011). In particular, skin-to-skin contact may have a crucial significance in very early infancy. Indeed, developmental studies with preterm infants showed that skin-to-skin contact has long-lasting positive effects on physical growth, physiological regulation, and cognitive development (Field, 1998; Feldman et al., 2010) suggesting the critical involvement of bodily contact in shaping development trajectories. Thus, by manipulating the source of touch, we hypothesized that infants would specifically rely on affective touch derived via skin-to-skin contact, as this touch would consists in a combination of perceptive properties (e.g., texture, temperature, and mechanical characteristics) that ensure ecological validity and may convey affective and emotional valence critical for the development of bodily self-awareness. More specifically, we hypothesized an interaction effect between the activation of C-tactile system and the source of touch indicating that skin-to-skin contact may maximize the socio-affective meaning of slow tactile interaction. Alternatively, it is also possible that the detection of visual–tactile body-related synchrony is primarily modulated by interoceptive signals related to the activation of C-tactile system (solely based on velocity properties of tactile stimulation) and independently from the source of touch.

## Materials and Methods

### Participants

The study was conducted at the Pediatric Unit of Monfalcone Hospital (GO, Italy) where infants were born. Fifty-five-month-old infants (19 female and 31 male; mean age 153.08 days) at time of test took part in the study. Thirteen additional infants participated but were excluded due to strong side bias (e.g., they looked >85% of the time to the same side of the screen, *N* = 4) or because they failed to complete the task due to fussiness (*N* = 9). All infants met the screening criteria for normal delivery: gestational age > 37 weeks, birth weight > 2500 g, Apgar score ≥ 8 at 5 min after birth. All infants were Caucasian. After been informed about the procedure, parents gave informed consent for their child’s participation. The local Ethical Committee of Psychological Research (University of Padova) approved the study protocol.

### Stimuli and Procedure

**FIGURE 1:**
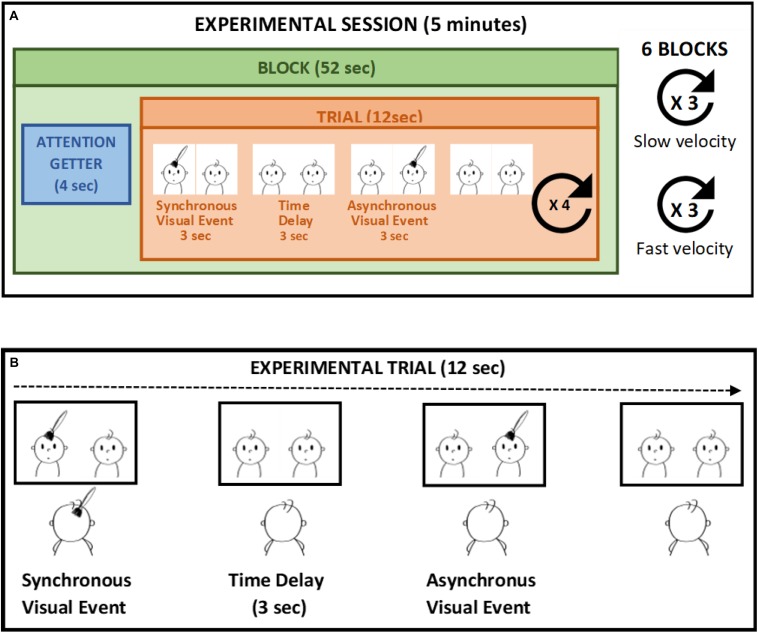
Example of experimental session **(A)**. In each trial **(B)** the tactile stimulation is applied in synchrony to the left visual stimulus (synchronous video) whereas the visual stimulus on the right is delayed (asynchronous video) in respect to the tactile stimulation. The touch velocity is manipulated whiting subjects, whereas the source of touch is manipulated as between-subjects variable.

The study took place in a dimly lit room within the hospital when the infants were in an alert and calm state. Infants sat on a car seat, at a distance of approximately 50 cm from the computer monitor. The screen was inclined to be parallel to the infants’ face and infants’ eye level was aligned with the center of the screen. A video camera mounted above the monitor and centered on the infants’ face was used to record the infants’ gaze and eye movement. Infants were presented with two side-by side and previously recorded videos of a 5-month-old infant’s face been touched on the forehead every 12 s. The tactile stimulation presented on the screen lasted 3 s. The two videos were identical, except from the fact that one of them was time delayed by 6 s compared to the other. So that, while one video displayed the touch at the beginning of the trial, in the other video the same tactile event occurred after 6 s. During the video presentation infants were also touched on their own forehead in a way that matched the video presentation in terms of spatial position, movement direction stroking velocity, and source of touch. An experimenter who stood behind the infant to prevent them from being distracted delivered stroking manually (Filippetti et al., 2013, 2015). Given the time delay of 6 s between the two side-by-side videos, the tactile stimulation felt by the infants was delivered synchronously with respect to one video display and asynchronously with respect to the other. The time windows of the stimuli was selected in order to ensure a delay of 3 s between the end of the synchronous visual stimulus and the beginning of the asynchronous visual stimulus, as previous studies demonstrated that a 3 s time-delay prevents detecting the contingency between two sensory events in infants younger than 6 months of age (Gergely and Watson, 1999). Each trial lasted 12 s and comprised of a synchronous video stimulus, whereby the tactile event displayed on the screen was contingent to the tactile stimulation on the infant’s forehead, and an asynchronous video stimulus, during which the tactile event displayed on the screen was delayed by 3 s with respect to the tactile stimulation. Each infant was presented with 24 trials divided in six blocks of four trials. A brief attention getter lasting 4 s (i.e., a colorful cartoon with sound) was presented before each block in order to keep the infants’ attention on the screen. We manipulated the touch velocity as a within-subjects variable, presenting the infants with a slow touch delivered at 3 cm/s, which falls into the optimal velocity range for activating C-tactile fibers, and a fast touch delivered at 18 cm/s. The position of the synchronous video (left and right) and the touch velocity were randomized between blocks (three blocks for a total of 12 trials for slow velocity and three blocks for fast velocity). Moreover, we manipulated the source of touch as a between-subjects variable. That is, infants were randomly assigned to two different groups (25 participants in each group) and one group of infants was stroked by a human hand while another group was touched with a brush (Figure 1).

### Data Analysis

Looking behavior toward the stimuli was recorded and off-line coded to calculate the cumulative looking time to both synchronous and asynchronous videos for each trial. A trial was considered valid only if the infant attended to both the synchronous and the asynchronous visual stimuli for at least 200 ms during the critical time window in which the tactile interaction was displayed on the screen. We included in the data analysis all participants who completed at least two valid trials in each experimental condition. For each valid trial we calculated the looking time toward the synchronous video, the looking time toward the asynchronous video and the total looking time (i.e., sum of the looking time to the synchronous and to the asynchronous videos). Then, a synchronous preference score was calculated considering the looking time to the synchronous video over the total looking time to the screen. This score was calculated on the looking time instead of the total time of stimulus presentation (12 s for each trial) in order to take into account the effective interest of the infant during each trial (Turati et al., 2005; Filippetti et al., 2016). The same experimenter coded all the videos using the Datavyu software, a video coding and data visualization tool for collecting behavioral data from video (Datavyu Team, 2014). A second independent observer, blind to the experimental hypothesis, performed off-line coding of a randomly selected subgroup of participants (eight subjects). Inter-rater reliability was found to be excellent (Hallgren, 2012). Inter-class correlation was calculated on the synchronous score (ICC = 0.916) and on both the looking time to the synchronous video (ICC = 0.965) and the asynchronous video (ICC = 0.809).

## Results

**FIGURE 2:**
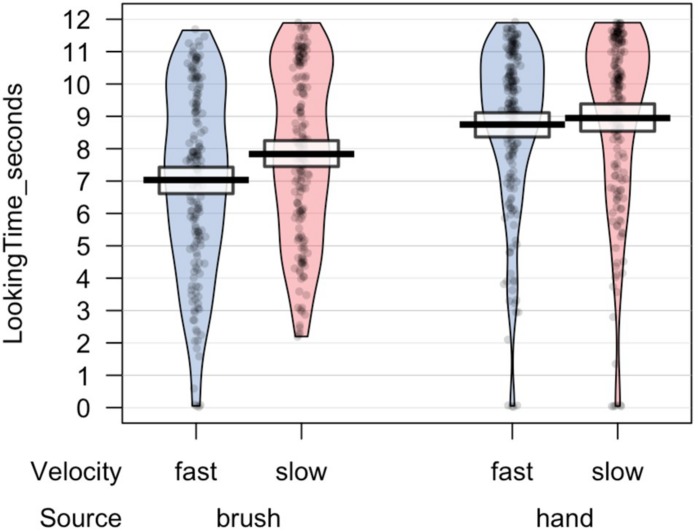
Total looking time toward the screen for each trial.

All statistical analyses were performed using R, a software environment for statistical computing and graphics (R Core Team, 2013). Preliminary analyses were performed to investigate the number of valid trials and the total looking time at the screen for each experimental condition. ANOVA mixed model was performed on the data in the long form, considering source of touch (hand vs. brush), touch velocity (slow vs. fast), and their interaction as fixed factors and participant ^∗^ velocity as random effect. The choice of using a mixed-effects model was determined by the possibility to take into account-fixed effects, which are parameters associated with an entire population, and random effects, which are associated with individual experimental units randomly drawn from population (Gelman and Hill, 2007). The results revealed no difference in the number of valid trials. Infants in the Hand Group completed on average 7.0 (SD = 3.09) valid trials for the slow velocity and 7.6 (SD = 2.69) valid trials for the fast velocity; infants in the Brush Group completed on average 6.72 (SD = 2.81) valid trials for the slow velocity and 6.80 (SD = 2.84) valid trials for the fast velocity. Analysis on the total looking time revealed a main effect of source of touch, *F*(1,46.4) = 11.739, *p* = 0.001, marginal *R*^2^ = 0.09, conditional *R*^2^ = 0.44, indicating that infants that experienced and saw a touch performed by a human hand looked longer at the videos, independently of the synchronicity between the visual and the tactile stimuli (Figure 2).

The main analyses were performed on the synchronous preference scores. Descriptive analysis revealed that on average infants in the Hand Group looked at the synchronous video for 48.65% (SD 21.03) of the looking time when the touch was delivered at slow velocity and for 48.64% (SD 17.18) when the touch was delivered at fast velocity; whereas infants in the Brush Group looked at the synchronous video for 55.48% (SD 23.06) of the looking time when the touch was delivered at slow velocity and for 49.86% (SD 20.92) when the touch was delivered at fast velocity. We wanted to explore infants’ visual preference asking whether infants showed a preference for the synchronous video. Simple *t*-test comparing the synchronous preference score with chance level (50%) were separately performed for each experimental condition. The results revealed that only infants touched with the brush at CT-optimal velocity looked longer at the synchronous video compared to the asynchronous, *T*(169) = 3.081, *p* = 0.002, *d* = 0.238; *p*-value adjusted for multiple comparisons using Bonferroni correction, *p* < 0.0125 (Table 1).

**TABLE 1:** Descriptive statistics for each experimental condition (mean and standard deviation of looking time in milliseconds and percentage over the total time of stimuli presentations −12,000 ms); synchronous preference score (percentage of looking time to the synchronous video over the total looking time); and simple *T*-test comparing the visual preference score with the chance level (50%).

**Group**	**Touch Velocity**	**Number of valid trials**	**Total looking time (ms)**	**Synchronous looking time (ms)**	**Asynchronous looking time (ms)**	**Synchronous preference score**	**Simple *T*-test (chance level)**
Hand	Slow	7.0 (3.84)	9106 (2385) 76%	4422 (2298) 37%	4684 (2287) 39%	48.65% (21.03)	*t* = −0.850 *p* = 0.397
	Fast	7.6 (2.69)	8869 (2271) 74%	4328 (1907) 36%	4541 (1900) 38%	48.64% (17.18)	*t* = −1.095 *p* = 0.275
Brush	Slow	6.7 (2.81)	7835 (2666) 65%	4335 (2392) 36%	3500 (2241) 29%	55.48% (23.06)	*t* = 3.081 *p* = 0.002
	Fast	6.8 (2.84)	7127 (2694) 59%	3526 (1991) 29%	3601 (2081) 30%	49.86% (20.92)	*t* = −0.085 *p* = 0.932

**FIGURE 3:**
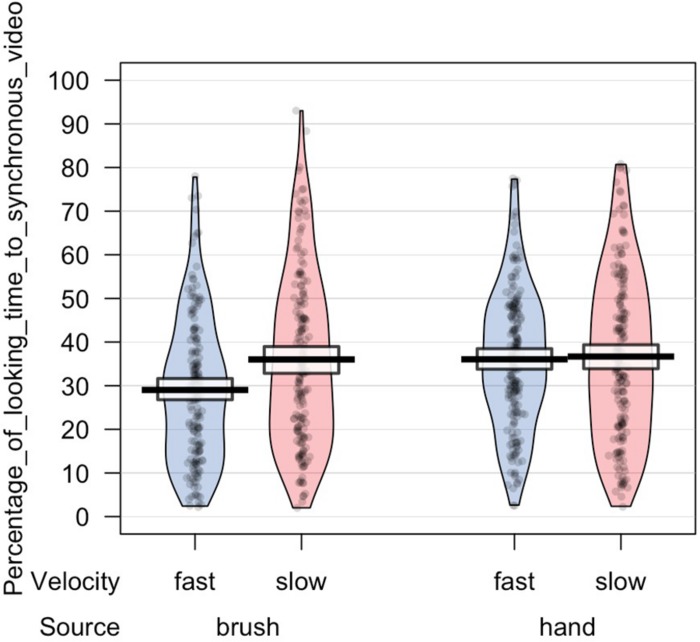
Percentage of looking time toward the synchronous video in each experimental condition.

**FIGURE 4:**
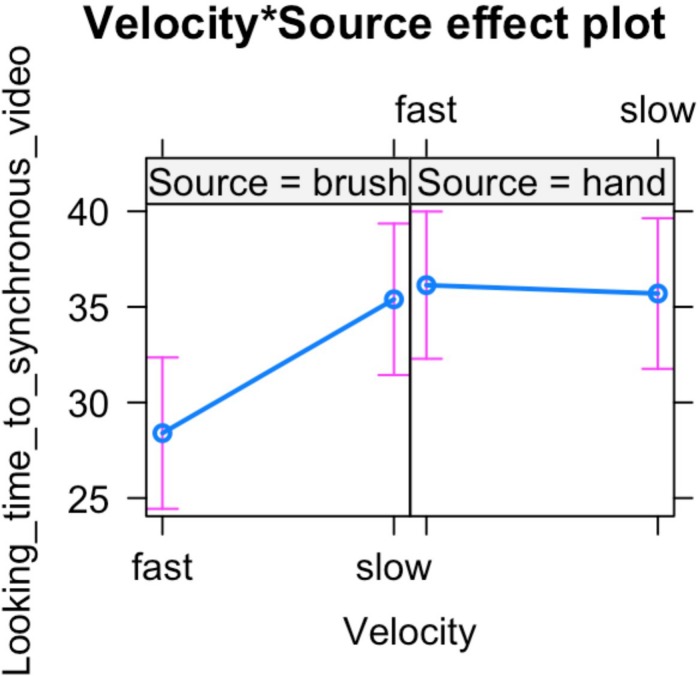
Percentage of looking time toward the synchronous video over the total time of stimulus presentation. Mean and standard errors are displayed for each experimental condition. The plot shows the interaction effect between velocity and source of touch.

In light of this result, we further analyzed infants’ looking time toward the synchronous video. ANOVA mixed model was performed on the data in the long form including all the valid trials completed by each participant in order to investigate the effect of source of touch (hand vs. brush) and touch velocity (fast vs. CT-optimal) on infants’ preference for the synchronous visual–tactile stimulation. We used a mixed-effects model to take into account random-effect factors (participant ^∗^ velocity) and to control for uneven number of observations. In our experiment, we excluded trials in which infant was not paying attention to the screen (e.g., looking away) resulting in a different number of trials for each participant (uneven observations). Thus, one advantage of using mixed models approach is that we can use all the data we have (i.e., data in the long form considering the full data set without averaging for condition) and missing scores have no effect on others scores from the same participant. We tested the full mixed-effects model including source of touch, velocity, and their interaction as fixed factors and participant ^∗^ velocity as random factor. The results revealed a significant interaction between source and velocity of the tactile stimulation, *F*(1,63.313) = 4.217, *p* = 0.044, marginal *R*^2^ = 0.03, conditional *R*^2^ = 0.20 (Figures 3, 4).

## Discussion

In the present study we investigated the role of affective touch in modulating infants’ ability to detect visual–tactile body-related synchrony. We manipulated the affective valence of the tactile stimulation by controlling for touch velocity, linked to the neurophysiological properties of the C-tactile system, and source of the touch, which refers to socio-affective meaning of the tactile interaction. First, in line with interoceptive theories (Fotopoulou and Tsakiris, 2017; Crucianelli and Filippetti, 2018) we predicted that the slow velocity represents an interoceptive dimension that can facilitate multisensory body-related perception. As such, infants in our study would display a visual preference for the synchronous compared to asynchronous visual–tactile stimulation only in the slow-touch condition. Second, based on developmental studies on the importance of skin-to-skin contact for conveying affective and emotional valence (Field, 1998; Feldman, 2011; Tuulari et al., 2019), we hypothesized that the perceptive properties of skin-to-skin contact would maximize the socio-affective meaning of tactile interaction, thus modulating infants’ preference for visual–tactile bodily synchrony.

The results are partially in line with our initial hypothesis as we found some expected as well as some unexpected results. With regard to the role of the C-tactile system in body perception, the results are in line with our hypothesis suggesting that in the brush condition the tactile velocity may have a role in modulating infants’ ability to detect visual–tactile body-related synchrony. When controlling for touch velocity, infants preferred slow touch applied in synchrony. While previous studies showed evidence of infants’ ability to detect multisensory synchrony without taking into account the affective aspects of tactile stimulation (Zmyj et al., 2011; Filippetti et al., 2013, 2016), in the present study we further demonstrate that infants’ visual preference for visual–tactile synchronous stimulation is specifically constraint by the affective properties of touch. This suggests that manipulation of affective touch modulates infants’ preference for bodily visual–tactile synchrony. Importantly, for the first time this result shows that slow touch might help infants to detect body-related visual–tactile synchrony, suggesting that interoceptive bodily signals may play a crucial role in the formation and modulation of bodily self-awareness. Instead, and contrary to our hypothesis, we did not find a significant effect of the hand condition. More specifically, we found an interaction effect between velocity and source of touch indicating that the two groups of infants (infants that were stroked with a human hand vs. infants that were stroked with a brush) showed a different visual behavior. In particular, our results revealed that infants that watched and experienced brush stroking at slow velocity displayed a preference for the visual–tactile synchronous video, compared to when the touch was delivered at fast velocity. Conversely, infants that were presented with a visual–tactile stimulus delivered by a human hand didn’t show an effect of touch velocity on visual behavior. Considering these results together, it is possible to speculate that infants’ early ability to detect visual–tactile body-related synchrony is primarily modulated by interoceptive signals conveyed by slow touch via the activation of C-tactile system, independently from the source of touch. This might suggest that information about the internal condition of the body (i.e., conveyed through the affective properties of touch in our study) promotes the development of bodily self-awareness, whereas the source of touch may modulate different information processing, such as its social properties (e.g., increasing attention to contingent social information; Della Longa et al., 2019). In particular, in the present study the slow touch in the brush condition may have helped infants in detecting visual–tactile synchrony whereas the social valence of skin to skin contact may have played a different role in capturing infant’s visual attention to the hand gestures. While this remains a tentative speculation, future studies should experimentally examine this possibility.

Our results are in line with developmental studies that show infants’ behavioral, physiological, and neural sensitivity to affective touch. Indeed, affective touch has been shown to be effective in reducing infants’ responses to stress (Stack and Muir, 1992), promote physical and neuro-cognitive development (Feldman et al., 2014), and modulate physiological state (Fairhurst et al., 2014; Aguirre et al., 2019). Moreover, a recent study reported activation of insular cortex in response to affective touch from 2 months of life (Jönsson et al., 2018) suggesting that infants are sensitive to interoceptive properties of affective touch from the earlies stages of life. However, other evidence suggest that the specialization of cortical processing of affective touch might still be ongoing during early infancy (Kida and Shinohara, 2013; Miguel et al., 2017; Pirazzoli et al., 2019). Thus, it is possible that while cortical responses to touch velocity, which selectively activate the C-tactile system, are already evident soon after birth (Jönsson et al., 2018), sensitivity to other perceptual properties that convey specific information about human contact, such as texture, body, and temperature, require more time to develop (Pirazzoli et al., 2019). According to a neurocostructivism perspective, early interoceptive sensitivity to affective touch may undergo a gradual process of functional specialization and cortical localization that provide the neurophysiological foundation for the emergence of socio-affective meaning of interpersonal contact (Johnson, 2001, 2011). This mechanism may be critically involved in the formation and maintenance of affiliative behaviors and social bonds (Morrison et al., 2010). If so, perceptual properties of tactile interaction experienced in conjunction with other multisensory social information (e.g., someone looking and talking to the infant while caressing her) may gradually acquire socio-affective valence and contribute in shaping socio-cognitive developmental trajectories. Indeed, 9-month-old infants have been shown to modulate their cardiac response to affective touch not just on the basis of mechanical properties but also according to its social source. Specifically, infants’ heart rate decreased more in response to stroke when their parent rather than the experimenter was present and this effect was found only for CT-optimal velocity (Aguirre et al., 2019). These findings may suggest that 9-month-old infants’ ability to respond to affective touch, based on the activation of the C-tactile system, support affective-motivational processing of tactile stimulation, and particularly so in socio relevant context. Overall, our results are in line with the neurocostructivism perspective as they show that early on, infants’ sensitivity in response to affective touch is based on the touch velocity when controlling for the source of the tactile stimulus, as reflected by a modulation of visual attention toward visual–tactile synchrony during slow touch with a brush. Conversely, infants presented with the hand condition showed a similar visual interest for both synchronous and asynchronous videos independently from touch velocity, suggesting that at 4 months of age hand gestures represent a salient stimulus that capture infants’ visual attention irrespectively of tactile information. Further investigation should consider different developmental ages in order to investigate whether later on, infants would specifically rely on affective and emotional valence of touch derived via skin-to-skin contact for the development of bodily self-awareness and the detection of visual–tactile synchrony.

When we consider the total looking time, infants in the hand group showed an overall longer looking to the video screen compared to infants presented with brush stimulation. The overall increase in looking behavior was irrespective of multisensory synchrony. This unexpected finding suggests that infants displayed a particular interest in looking at movements performed by a human hand independently of the matching with contingent tactile stimulation. A possible interpretation of this result can be found in the salience of human hand gestures. Different studies demonstrate infants’ early ability to detect biological movement (Fox and McDaniels, 1982; Bertenthal et al., 1984). More specifically, newborns can discriminate between possible and impossible dynamic hand gestures (Longhi et al., 2015) and are able to discriminate gestures that involve hand-to-hand touch, while they fail to discriminate the same interaction between an object and a hand (Addabbo et al., 2015). Moreover, 3-month-old infants display spontaneous preference for touching hand-to-face gestures compared to no-touching gesture, thus showing evidence of an early ability to recognize and prefer touching gestures involving the interaction between human body parts (Addabbo et al., 2015). Therefore, it could be speculated that early in life, hand gestures represent a relevant stimulus that can capture infants’ visual attention irrespectively of contingent tactile stimulation. This finding suggests that infants’ visual behavior may be modulated in a subtle manner by affective properties of the tactile stimulation as well as by visual information, which differently contribute in driving infants’ attention. Future studies may consider to control for the visual information by using an abstract representation indicating stroking that displays the same visual stimulus in both the tactile conditions.

One might wonder why we could only replicate the effect of visual–tactile synchrony only in the brush condition, but not in the hand condition. From a methodological perspective, one explanation might lie on the length of the stimuli, the presence of movement and the visual difference between stimuli. Compared to previous studies (Filippetti et al., 2016), a crucial difference in our paradigm is that, in order to take into account the stroking velocity of slow vs. fast touch, we had to extend the length of our tactile events. Consequently, infants had more time to direct their attention, explore the stimuli, and disengage before a new tactile event appeared on the other side of the screen. Moreover, the presence of movement and the salience of the visual stimulus may have played a predominant role on infants’ visual behavior. According to this interpretation, when the visual stimulus was particularly salient (human hand touch) infants looked longer to the screen irrespectively of the synchrony with the tactile stimulation and the velocity to which the touch was performed. Therefore, in the hand condition the absence of a visual preference for the synchronous video could be due to a celling effect in the looking time toward biological movement. Recent studies indicate that in adults the visual appearance of the own arm modulates the perceived pleasantness of touch (Keizer et al., 2019); moreover, cortical responses to vicarious tactile interactions are already present by 4-month of age (Rigato et al., 2019). These results point out the importance of the visual context in modulating responses to tactile stimulation. In the present study, infants’ visual behavior was shown to be differently modulated by the tactile information when infants were looking at biological movement compare to when they were looking to movement of an inanimate object. This suggest that there is a difference in how infants process skin to skin vs. object contact; however, the paradigm that we used in the present study cannot differentiate between the separate role of visual and the tactile information. Future investigations should be done to better understand the interplay between touch and vision in multisensory integration of bodily signals, taking into account the length and the salience of the visual stimuli.

## Conclusion

In conclusion, for the first time this study shows that affective touch may have a fundamental role in the development of bodily self-awareness in early infancy, as reflected by their ability to detect contingency between visual and tactile body-related stimulation. These findings pave the way for new perspectives for future research, showing that infants’ early sensitivity to affective touch may have a crucial role in the acquisition of body awareness and in distinguishing oneself from others with cascading effects on interpersonal engagement and social cognition abilities.

## Data Availability Statement

The raw data supporting the conclusions of this article will be made available by the authors, without undue reservation, to any qualified researcher.

## Ethics Statement

The studies involving human participants were reviewed and approved by the Comitato Etico della Ricerca Psicologica Area 17 Dipartimenti/Sezione di Psicologia Università degli Studi di Padova. Written informed consent to participate in this study was provided by the participants’ legal guardian/next of kin.

## Author Contributions

LD, MF, and TF discussed the project, developed the hypothesis, and designed the method. LD prepared the materials. DD contributed to the provision of the resources (laboratory spaces and participants). LD collected and analyzed the data. TF and DD supervised the data collection. LD and MF prepared the manuscript. All authors approved the final version of the manuscript.

## Conflict of Interest

The authors declare that the research was conducted in the absence of any commercial or financial relationships that could be construed as a potential conflict of interest.
